# The Women4Health cohort: a unique cohort to study women-specific mechanisms of cardio-metabolic regulation

**DOI:** 10.1093/ehjopen/oeae012

**Published:** 2024-02-27

**Authors:** Fabio Busonero, Stefania Lenarduzzi, Francesca Crobu, Roberta Marie Gentile, Andrea Carta, Francesco Cracco, Andrea Maschio, Silvia Camarda, Michele Marongiu, Daniela Zanetti, Claudio Conversano, Giovanni Di Lorenzo, Daniela Mazzà, Francesco De Seta, Giorgia Girotto, Serena Sanna

**Affiliations:** Institute of Genetic and Biomedical Research (IRGB), National Research Council (CNR), c/o Cittadella Universitaria di Monserrato, SS554 Km 4500, Monserrato, 09042, CA, Italy; Institute for Maternal and Child Health—IRCCS ‘Burlo Garofolo’, Via dell'Istria 65/1, Trieste, 34137, TS, Italy; Institute of Genetic and Biomedical Research (IRGB), National Research Council (CNR), c/o Cittadella Universitaria di Monserrato, SS554 Km 4500, Monserrato, 09042, CA, Italy; Department of Medicine, Surgery and Health Sciences, University of Trieste, Piazzale Europa 1, Trieste, 34137, TS, Italy; Department of Business and Economics, University of Cagliari, via Università 40, 09124, Cagliari, CA, Italy; Department of Medicine, Surgery and Health Sciences, University of Trieste, Piazzale Europa 1, Trieste, 34137, TS, Italy; Institute of Genetic and Biomedical Research (IRGB), National Research Council (CNR), c/o Cittadella Universitaria di Monserrato, SS554 Km 4500, Monserrato, 09042, CA, Italy; Department of Medicine, Surgery and Health Sciences, University of Trieste, Piazzale Europa 1, Trieste, 34137, TS, Italy; Institute of Genetic and Biomedical Research (IRGB), National Research Council (CNR), c/o Cittadella Universitaria di Monserrato, SS554 Km 4500, Monserrato, 09042, CA, Italy; Institute of Genetic and Biomedical Research (IRGB), National Research Council (CNR), c/o Cittadella Universitaria di Monserrato, SS554 Km 4500, Monserrato, 09042, CA, Italy; Institute of Genetic and Biomedical Research (IRGB), National Research Council (CNR), c/o Cittadella Universitaria di Monserrato, SS554 Km 4500, Monserrato, 09042, CA, Italy; Department of Business and Economics, University of Cagliari, via Università 40, 09124, Cagliari, CA, Italy; Institute for Maternal and Child Health—IRCCS ‘Burlo Garofolo’, Via dell'Istria 65/1, Trieste, 34137, TS, Italy; Institute for Maternal and Child Health—IRCCS ‘Burlo Garofolo’, Via dell'Istria 65/1, Trieste, 34137, TS, Italy; Institute for Maternal and Child Health—IRCCS ‘Burlo Garofolo’, Via dell'Istria 65/1, Trieste, 34137, TS, Italy; Institute for Maternal and Child Health—IRCCS ‘Burlo Garofolo’, Via dell'Istria 65/1, Trieste, 34137, TS, Italy; Department of Medicine, Surgery and Health Sciences, University of Trieste, Piazzale Europa 1, Trieste, 34137, TS, Italy; Institute of Genetic and Biomedical Research (IRGB), National Research Council (CNR), c/o Cittadella Universitaria di Monserrato, SS554 Km 4500, Monserrato, 09042, CA, Italy; Department of Genetics, University Medical Center Groningen, Hanzeplein 1, 97123 GZ, Groningen, The Netherlands

**Keywords:** Women, Cardiovascular diseases, Lipids, Sex hormones, Microbiome, Multi-omics study

## Abstract

**Aims:**

Epidemiological research has shown relevant differences between sexes in clinical manifestations, severity, and progression of cardiovascular and metabolic disorders. To date, the mechanisms underlying these differences remain unknown. Given the rising incidence of such diseases, gender-specific research on established and emerging risk factors, such as dysfunction of glycaemic and/or lipid metabolism, of sex hormones and of gut microbiome, is of paramount importance. The relationships between sex hormones, gut microbiome, and host glycaemic and/or lipid metabolism are largely unknown even in the homoeostasis status. Yet this knowledge gap would be pivotal to pinpoint to key mechanisms that are likely to be disrupted in disease context.

**Methods and results:**

Here we present the Women4Health (W4H) cohort, a unique cohort comprising up to 300 healthy women followed up during a natural menstrual cycle, set up with the primary goal to investigate the combined role of sex hormones and gut microbiota variations in regulating host lipid and glucose metabolism during homoeostasis, using a multi-omics strategy. Additionally, the W4H cohort will take into consideration another ecosystem that is unique to women, the vaginal microbiome, investigating its interaction with gut microbiome and exploring—for the first time—its role in cardiometabolic disorders.

**Conclusion:**

The W4H cohort study lays a foundation for improving current knowledge of women-specific mechanisms in cardiometabolic regulation. It aspires to transform insights on host–microbiota interactions into prevention and therapeutic approaches for personalized health care.

## Introduction

Cardiometabolic disorders (CMD), a group of conditions that include cardiovascular diseases (CVD), type 2 diabetes (T2D) and metabolic syndrome, are characterized by dysfunction of glycaemic and/or lipid metabolism. They are a leading cause of morbidity and mortality worldwide, with a death rate that is constantly increasing. The complexity of CMD is amplified by relevant differences in clinical manifestations, severity, and progression between sexes,^1–3^ the underlying mechanisms of which are largely unknown. Prognosis is poorer in women—for example, despite the higher age-standardized incidence of CVD observed in men than women, in Europe, CVD accounted for 39% of all deaths in men and 46% of all deaths in women.^4^ Therefore, there is a global, urgent need for better women’s tailored prevention and treatment strategies.^5^

Genetic, intrinsic and extrinsic factors contribute to CMD, nonetheless female-specific mechanisms of CMD and their risk factors are only minimally explained by genetics.^6–9^ Among the extrinsic factors associated with CMD and their risk factor, particular attention has been given recently to the gut microbiota, the community of microbes inhabiting the gastrointestinal tract. In both humans and mice, significant imbalance of the gut microbiome composition and function (dysbiosis), and in gut microbiota-produced metabolites measured in faeces, has been associated with CMD and increased CMD-related risk factors,^10–19^ including insulin sensitivity and T2D,^20,21^ cholesterol levels,^12,22,23^ and atherosclerosis.^13^ There is also emerging evidence that female sex hormones could directly influence the gut microbiome: studies in humans and mice showed that large hormonal changes, such as those occurring during menopause and pregnancy, are associated with reduced species richness and diversity of the gut microbiome.^24–26^ It is thus plausible to think that large variations in female’s sex hormones, impacting on the gut microbiome, could explain the sex dimorphism in CMD.

Conversely, it is understudied if small variation in sex hormones during homoeostasis, e.g. those occurring regularly during the menstrual cycle, could influence gut microbiome. A recent study carried out in 20 women found nominal evidence for a role of progesterone in influencing temporal microbiota variation.^27^ While the association was not significant after multiple testing, the observation remained inconclusive not only for the small sample size but also for the reduced precision on sex hormones values, which were inferred based on the day of the menstrual cycle rather than being directly measured. Previous studies on female’s health have shown that variations in sex hormones during the menstrual cycle are significantly associated with changes in CMD-risk factors, specifically lipid levels, insulin resistance, and transaminases.^28–30^ Therefore, the menstrual cycle offers a natural endeavour to study simultaneous variation of sex hormones, gut microbiome, and CMD-related metabolism, without the need of any intervention to modulate sex hormones.

Here we present the Women4Health (W4H) cohort, an observational study of 300 women during a natural menstrual cycle. The study aims to investigate for the first time the hypothesis that the cross-talk between sex hormones and gut microbiome results in differential host metabolism regulation in females also during homoeostasis and in the reproductive age.

A multi-omics strategy, including genomic, metagenomic, metabolomic, and proteomic approaches, will be used to characterize biological and physiological mechanisms.

The other unique feature of the W4H study is the collection of bio-samples to investigate the vaginal microbiome in the contest of cardiometabolic regulation. While vaginal dysbiosis has been associated with gynaecological disorders (such as bacterial vaginosis, higher predisposition to local infections, miscarriages, and preterm births),^31–33^ it has never been explored whether the resulting local inflammation can perturbate nearby organs, including the gut, thus potentially affecting gut microbiome composition and function and its role in regulating host metabolism.

Here we describe the W4H cohort’s study protocol and its unique (bio)data collection.

## Methods

### Study organization

Women4Health is a study cohort created by a joint collaborative effort between the women’s and children’s hospital IRCCS Burlo-Garofolo in Trieste, Italy, (P.I.: Prof. Giorgia Girotto) and the Institute for Genetics and Biomedical Research of the National Research Council (IRGB-CNR) in Cagliari, Italy (P.I.: Dr. Serena Sanna). The study was designed by Dr. Serena Sanna (IRGB-CNR), and fundings were gathered by her and by Prof. Giorgia Girotto (see ‘Funding’ statement). The protocol was designed by researchers from both Institutes (see ‘Authors contribution’ section). Daily management of volunteers’ recruitment, collection of informed consent forms and biological specimens, measurement of sex hormones, and blood biochemistry parameters is performed by the IRCC Burlo-Garofolo team. All biological samples are then shipped to IRGB-CNR for long-term storage and processing. Database management and statistical data analysis are also performed by IRGB-CNR researchers. Project advertising and communication activities are carried out by several members of both research groups.

### Study design

**Figure 1:**
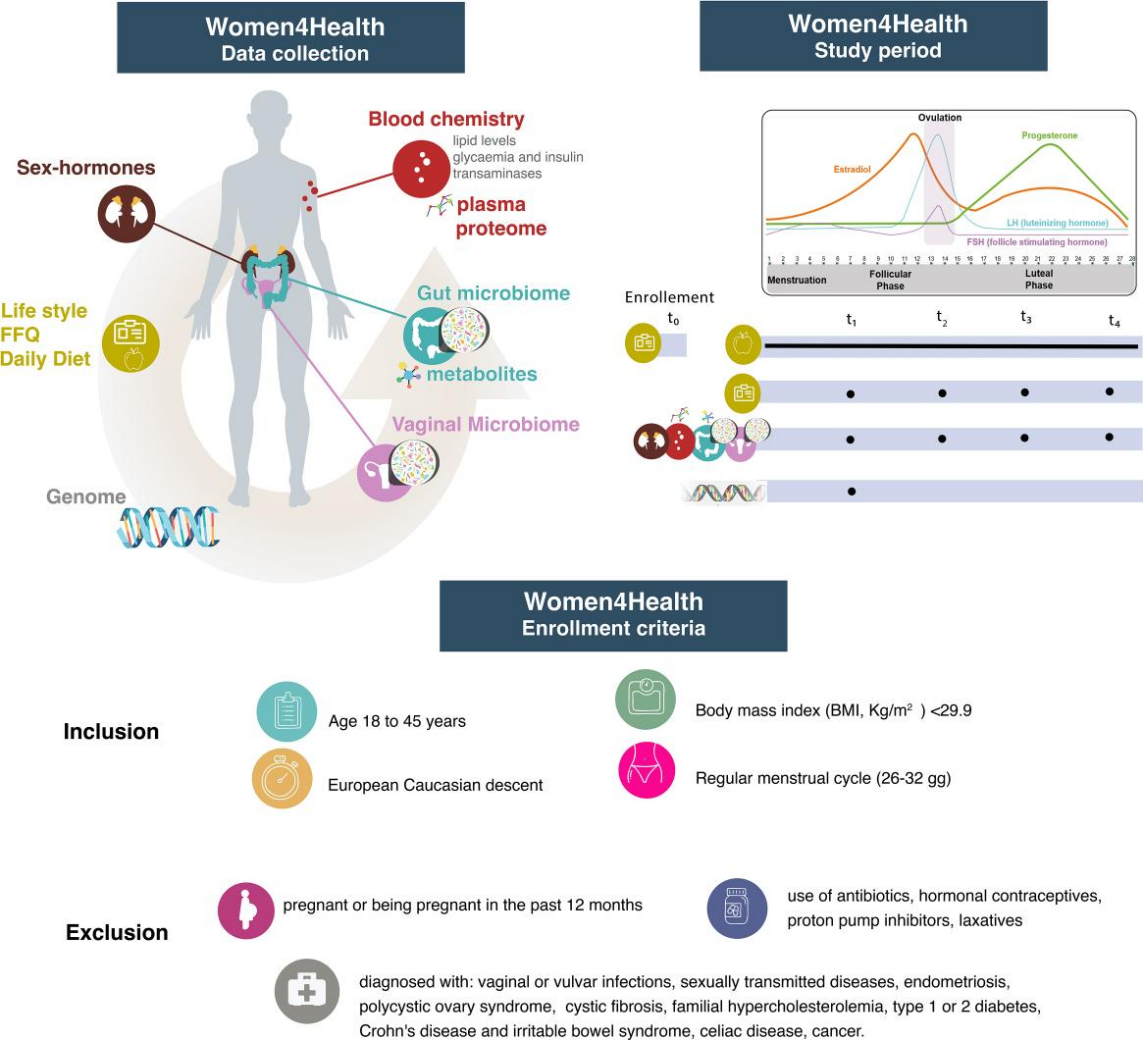
Scheme of the study design. Schematic representation of study design, showing data collection, study period, and enrolment criteria. The arrow in the first panel points to phenotypes considered as outcomes. The figure was designed using infographics downloaded from Freepik.com.

The Women4Health study aims to recruit 300 women; recruitment is currently carried out at the women’s and children’s hospital IRCCS Burlo-Garofolo in Trieste (Italy), according to the protocol described below and depicted in *Figure 1*.

Upon inclusion and signature of informed consent, volunteers are enrolled in the study and information on family history, clinical and anthropometric data, lifestyle, and diet information are collected via questionnaires. Volunteers are asked to record their daily diet and sport at the first day of the menstrual cycle and for up to 28 days or until the next menstrual cycle. They are then scheduled for follow-up at four different time points: the 1st at the follicular phase (between days 6 and 9), the 2nd at the ovulation phase (between Days 12 and 16), the 3rd at the early luteal phase (between Days 18 and 22), and the 4th at late luteal phase (between Days 24 and 28). Depending on the day at enrolment, volunteers can start at any of the follow-up visits and continue with the remaining points of observations at the next menstrual cycle. At these time points, two stool and one vaginal swab specimens are self-collected by volunteers at home, then handed at the Obstetric and Gynaecologic (ObG) Clinic of the IRCCS Burlo-Garofolo no later than 24 h after collection. On the day biospecimens are handed to the ObG Clinic, women undergo a blood withdrawal after overnight fasting and fill in questionnaires to report health status in the week preceding collection and information on collected biological samples. Furthermore, a saliva sample for DNA extraction is collected (on the first visit only).

### Consent

The study protocol—including informed consent forms—was approved by the Friuli-Venezia Giulia Ethical Committee on 09/2021 with prot. N. 0034184/P/GEN/ARCS and modifications amended on 05/09/2023.

### Eligibility criteria

The W4H study enrols women aged between 18 and 45 years old, an age range selected to avoid enrolling women in their pubertal or pre-menopausal phase. To avoid the introduction of confounding factors due to diseases or ongoing therapies, the cohort has been designed as follows: non-obese (BMI 17-29.9) women with a regular menstrual cycle (25–34 days), who have not made use in the previous 30 days of systemic or local hormonal contraceptives, antibiotics, proton-pump inhibitors, metformin, statin, and laxatives, and who do not have ongoing vaginal/vulvar infections or sexually transmitted diseases. Additionally, they must not have been diagnosed with CMD, gastrointestinal diseases, inherited metabolic disorders (cystic fibrosis), diabetes, familial hypercholesterolaemia and endometriosis or polycystic ovary syndrome, or have not been subjected to cancer therapy. Finally, we also exclude women who have been pregnant in the last 12 months. To assure genetic homogeneity across participants, we focus on women of European ancestry only, who are the majority of women living in the area of Trieste.

### Recruitment protocol

Potential participants are invited in the study via the ObG Clinic, via advertisement within the hospitals and research centres in Trieste, via announcements on the Women4Health social media pages (Facebook and Instagram), via local radio and press communications, and other outreach activities. A leaflet containing details about W4H study and the abovementioned criterion for enrolment is posted in our social media pages and distributed in the hospital halls, universities, and aggregation centres.

Women who contact the recruiting team via email are invited for a meeting at the ObG clinic of the IRCCS Burlo-Garofolo and informed about the purposes of the research project and protocol of the study. Those interested in study participation and who meet the recruitment criteria are asked to sign an informed consent form to (i) agree to participate in the study and (ii) for the collection of data and biological samples to be used for research purposes only. Each enrolled volunteer is assigned a numerical code and any pseudonymized participant’s information entered into the *ad hoc* created database. Participation is on a voluntary basis without any financial remuneration.

### Data collection and management

Data collection started in February 2022, after infection from COVID-19 started to decrease in incidence in Italy due to the vaccination campaign. At enrolment, women are asked to fill in an electronic questionnaire containing information about demographic and anthropometric measurements, detailed medical and gynaecological history, family disease history, direct or indirect exposure to smoke, use of pre-/pro-biotics, and dietary habits using a frequency food questionnaire validated in the Italian population.^34^ During each follow-up visit, another questionnaire is administered to collect information regarding weekly bowel movements and stool consistency using the Bristol scale charts,^31^ history of COVID-19 vaccination or infection, use of medications or supplements, and sexual activity in the week preceding the appointment. Questionnaires are filled in by volunteers on an electronic form created and managed using the Research Electronic Data Capture (RedCap) tools hosted at IRGB-CNR.^35,36^ REDCap is a secure, web-based software platform designed to support data collection for research studies, providing (1) an intuitive interface for data entry; (2) audit trails for tracking data manipulation and export procedures; (3) automated export procedures for seamless data downloads to common statistical packages; and (4) procedures for data integration and interoperability with external sources.

Daily diet and physical activity information are recorded by the volunteers themselves using MyFitnessPal, a smartphone app validated for scientific research studies.^27,37^ Daily diaries are shared by volunteers via email at the end of the observation period as PDF files.

A Data Management Plan has been set up and will be updated every 6 months.

### Collection, process, and storage of biological samples

Kits for self-collection of saliva, stool, and vaginal swabs are distributed to volunteers at enrolment along with proper instructions. To facilitate self-collection and assure robustness to room temperature, we use the following collection kits from DNA Genotek®: Oragene-DNA (OG-500) for saliva, OMNImet-GUT (ME-200) and OMNIgene-GUT (OM-200) bundle for faces, and OMNIgene-VAGINAL (OMR-130) for vaginal swabs. After collection, volunteers store and hand those at room temperature to the ObG Clinic within 24 h; collection kits are then promptly stored at the appropriate temperature, as recommended by the manufacturer. Blood is collected from venepunctures in silica gel-Vacutainer, and allowed to clot by leaving it undisturbed for 1 h at room temperature (RT), then 0.5 mL of serum, obtained by centrifugation at 4000 r.p.m. (equivalent to 2990 g) for 5 min at RT, is immediately transferred into two sterile safe-lock polypropylene tubes and stored at −20°C. Plasma is separated from whole blood and collected into citrate-treated tubes by centrifugation at 3000 r.p.m. (equivalent to 1680 g) for 10 min at RT. A total of 0.5 mL of plasma is then transferred into two sterile safe-lock polypropylene tubes and stored at −80°C. Serum is used to assess sexual hormones [progesterone, 17beta-oestradiol (17BE), follicle-stimulating hormone (FSH), luteinizing hormone (LH) and prolactin, fasting insulin, thyroid-stimulating hormone TSH, free thyroxine (FT4), and testosterone (last three only at first visit) using ADVIA Centaur CP Immunoassay System (Siemens Healthineers AG)]. An aliquot of serum is used to evaluate cardiometabolic profile [glycaemia, total-, high-density lipoprotein-, and low-density lipoprotein cholesterol (TC, HDL-C, and LDL-C, respectively), and triglycerides, as well as alanine aminotransferase (ALT) and aspartate aminotransferase (AST)] using VChemy S (Voden Medical Instrument SpA) and stored at IRCCS Burlo-Garofolo. All other biological samples (plasma, saliva, stool samples, and vaginal swabs) are shipped to the Institute for Genetic and Biomedical Research—National Research Council (IRGB-CNR) on a regular basis (every 21 days) in dry ice (stool in tube for metabolomics analyses and plasma aliquots), and at RT (saliva, vaginal samples, and stool in tube for DNA analyses). Once biological samples are received, human genomic DNA is extracted from saliva and stored at −20°C. Microbial DNA is isolated from stool (tubes for DNA analyses) and vaginal swabs, using standard protocols and DNA stored at −80°C.^38,39^ Aliquots of plasma and stool samples (tubes for metabolomic analysis) are instead directly stored at −80°C.

### Plans for omics data generation

Genomic, metagenomic, metabolomic, and proteomic approaches will be used to characterize biological and physiological mechanisms.

For DNA extracted from saliva samples, we will use whole-genome genotyping arrays coupled with genotype imputation to characterize the human genome.^40^ For DNA extracted from stool samples, whole-metagenomic shot-gun sequencing will be employed to achieve a taxonomic resolution down to species- and strains-level and to obtain functional information through microbial pathways definition. Furthermore, faecal metabolome will be derived using high-throughput technologies; the specific platform has not yet been identified. For DNA extracted from vaginal samples, 16S rRNA plus ITS amplicon sequencing will be instead used to determine taxonomic profiles (bacteria, archaea, and fungi composition) down to at the genus level.

Finally, the plasma proteome—including hundreds of proteins related to cardiovascular and metabolic functions—will be characterized using the Olink Proteomics platform.

### Statistical analysis

Descriptive statistics presented in this manuscript were derived using the R software for statistical computing (version 4.2.1).

For each sex hormone, we assessed the significance of overall changes across the four time points of collection using the non-parametric Friedman test for paired samples. Pairwise differences in trait means were assessed using the non-parametric Wilcoxon test for paired samples and derived Bonferroni adjusted *P*-values. In addition to the measured blood-chemistry traits, we also derived parameters of insulin resistance and beta-cell function using the Homoeostasis Model Assessment, as follows: HOMA-IR = [fasting insulin (µU/mL) × fasting plasma glucose (mg/dL)]/405; HOMA-B = 20 × fasting insulin (µU/mL)/[fasting plasma glucose (mg/dL) − 63].^41^

Code used for data clean-up, statistical analyses, and visualization of results is posted as soon as been validated in a freely available GitHub repository, at the following link: https://github.com/Sanna-s-LAB/Women4Health/.

### Recruitment, adherence rates, and basic descriptive statistics

From February 2022–August 2023, we enrolled 86 volunteers with an average of 4.7 volunteers per month. Among these, 3 have withdrawn their consent to the study before any follow-up visit and 59 engaged for at least one follow-up visit, resulting in an adherence rate of 69%. No difference in mean age was observed between volunteers that did not engage in any follow-up visits and those who returned for at least one (two-sided Wilcoxon-rank *P* = 0.93).

In total, 47 volunteers completed all four visits, while the remaining completed only three or less visits for different reasons: COVID-19 infection (4), menstruation arrived earlier (3), work-related issues (3), car accident (1), and issues on recording diet information (1). Therefore, sample size is different at each follow-up visit (59 samples at visit 1, 58 samples at visit 2, 53 samples at visit 3, 47 samples at visit 4).

Volunteers who engaged for at least one visit were aged 28 years on average (age range was from 21–40 years), and the average length of the menstrual cycle and menses were 28 days and 4.9 days, respectively (*Table 1*). All volunteers had sex hormonal levels within the expected range during reproductive age, and none showed evidence of endocrine diseases based on TSH, FT4, and testosterone levels measured at first visit.

**Table 1:** Descriptive statistics

Phenotype (unit)	Min–max	Mean (SD)
Age (years)	21–40	28.17 (5.59)
Average duration of menstruations (days)	3–7	4.95 (0.80)
Average duration of menstrual cycle (days)	23–34	28.26 (1.88)
Height (cm)	154–183	167.20 (6.30)
Weight (kg)	48.00–82.50	61.29 (9.44)
BMI (kg/m^2^)	17.71–29.23	21.09 (2.9)
TSH (µLU/mL)	0.07–6.33	1.28 (0.94)
FT4 (ng/dL)	0.54–1.78	1.01 (0.21)
Testosterone (ng/dL)	7.93–29.41	14.95 (5.10)
Glucose (mg/dL)	72–121	90.78 (7.77)
Insulin (mU/L)	0.8–14	4.40 (2.45)
HOMA-IR	0.17–3.30	1.01 (0.60)
HOMA-B	0.61–10.78	3.45 (2.15)
Total cholesterol (mg/dL)	137–268	188.8 (30.53)
HDL-C (mg/dL)	35–99	64.9 (13.07)
LDL-C (mg/dL)	52–191	109.1 (13.07)
Triglycerides (mg/dL)	33–145	75.14 (24.17)
AST (U/L)	12–43	18.66 (5.11)
ALT (U/L)	6–31	15.29 (5.14)

Descriptive statistics at enrolment for the 59 volunteers for whom at least one blood withdrawal is available. Statistics for phenotypes measured in blood are given only for the first visit.

BMI, body mass index; TSH, thyroid-stimulating hormone; FT4, free thyroxine; HDL-C, high-density lipoprotein cholesterol (mg/dL); LDL-C, low-density lipoprotein cholesterol; ALT, alanine transaminase (U/L); HOMA-IR, insulin resistance; HOMA-B, insulin sensitivity; SD, standard deviation.

**Figure 2:**
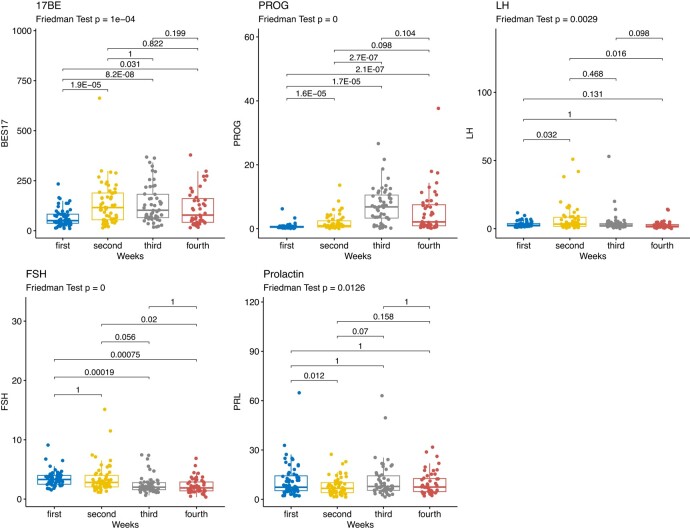
Variation of sex hormones by week of collection. Box plots describing the distribution of sex hormones at each time point. In each box plot, the tick line represents the median while the edges of the box represent the 25% and 75% of the distribution; whiskers represent 5% and 95% percentiles. For each trait, *P*-values of both the Friedman test for overall differences and the Wilcoxon test for pairwise differences are reported. 17BE, 17beta-oestradiol (pg/mL); PROG, progesterone (ng/mL); LH, luteinizing hormone (mIU/mL); FSH, follicular-stimulating hormone (mIU/mL); PRL, prolactin (ng/mL).

Distribution of sex hormones across time points reflect the expected variation during the menstrual cycle phases, with 17beta-oestradiol showing a sharp significant increase in the ovulation phases (second time point) and progesterone in the mid luteal phase (*Figure 2*).

## Discussion

The Women4Health is the first longitudinal study that focuses on the menstrual cycle to evaluate the impact of female sex hormonal variations on commensal bacteria and CMD-related markers. Mimicking a perturbation study by following up physiological hormonal changes during the menstrual cycle, invasive hormonal treatments necessary for an intervention study will be avoided. Compared to a cross-sectional study, the longitudinal design adds the unique opportunity to understand the dynamics and derive causal relationships between components under study. The longitudinal component is in fact extremely relevant in the metagenomic field to understand the causal role of microbiome in phenotypic changes, as well as disease onset and progression.^26,42,43^ Moreover, the W4H study has a short longitudinal observation time restricted to a maximum of 30 days per person, allowing for proper control in analyses of major confounders of the microbiome, such as diet and medication use. In contrast, since menopause is a long process lasting several years, a longitudinal study targeting hormonal changes in menopause would need to follow women for up to several years, making the collection and adjustment for confounding factors unfeasible.

Furthermore, by studying females in the homoeostasis status *bias from reverse causality* (diseases caused mechanisms) and *therapy-induced effects* are absent in the Women4Health cohort, biases that would instead be present in a *case–control study* of women in menopause with and without a diagnosis of CMD. Notably, studying the healthy population to learn potential mechanisms of diseases has been a successful strategy in genome-wide association studies. In fact, these have shown that genes associated with variation of quantitative traits in the general population harbour variants associated with both complex diseases and Mendelian disorders.^44–47^

Results from the W4H study fill in the current knowledge gap on female-specific mechanisms underlying cardiovascular and metabolic functions during homoeostasis and suggest putative targets to follow-up in designed cohorts of CMD patients. The identification of specific vaginal and gut microbiome features, associated with changes in host metabolism during homoeostasis and disease, will provide potential unexplored routes for prevention of CMD in women through screening of microbiome signatures and treatments through microbiome modulation. This will be of great worth considering that, despite the high mortality, CMD in women remains understudied, under-recognized, underdiagnosed, and undertreated and research studies oriented on the identification of sex-specific mechanisms and sex-specific risk factors underlying the pathophysiology of these disorders are recognized as a priority of the medical research field.^5^

Noteworthy, the W4H study has high potential to boost future female-specific research studies for other complex diseases. In fact, it will provide the first comprehensive evaluation of gut and vaginal microbiota variation within a menstrual cycle, filling an existing knowledge gap on sex hormones–microbiome interaction that is essential for understanding not only CMD, but potentially many other diseases with a clear sex dimorphism and for which a putative role of sex hormones and gut microbiome has been suggested.

The Women4Health cohort study also has challenges and limitations. The most compelling challenge is the recruitment of women who do not use local or systemic hormonal contraceptives. While in Italy the percentage of females using these medications is very low (20%) compared to other European countries (50% in North Europe)^48^ still this inclusion criteria needs to be added to others, such as absence of other diseases, pregnancy, as well as the determination of the volunteers to comply with a strict follow-up protocol of 30 days. The cohort is limited towards the characterization of cardiovascular biomarkers. While we measure lipids as the main risk factor for cardiovascular diseases, and hundreds of circulating proteins relevant for the cardiometabolic system, we do not have information on other relevant measurements of CVD risk, such as body fat composition, or measurements of cardiac health, such those that could be derived with an electrocardiogram. Future collaborative expansions of the Women4Health cohort will take this into consideration.

In conclusion, the unique design of Women4Health cohort study establishes the groundwork for improving current knowledge of women-specific mechanisms involved in cardiometabolic regulation. These insights have the potential to inform future translational applications aiming to convert gained knowledge on host–microbiota interactions into approaches for prevention and therapy through microbiota modulation, ultimately contributing to the development of personalized strategies.

## Data Availability

We are open for collaboration with research groups that would like to join this effort as an add-on using the infrastructure at IRGB-CNR and IRCCS Burlo-Garofolo or as a collaborative effort. Detailed information can be requested by email to the corresponding authors of this manuscript. Data for this study will be available after 2 years of the conclusion of the ERC project SEMICYCLE, which funds this effort (see ‘Funding’ statement).

## Lead author biography

Dr Serena Sanna is Director of Research and Principal Investigator at the Institute of Genetic and Biomedical Research of the CNR, Italy. She is an internationally recognized researcher in the field of genetic epidemiology of complex traits and diseases. With her group, her research focuses on understanding the role of the genome and human microbiome interaction in the regulation of health and diseases. Key components of her studies are special cohorts, cutting edge technologies, and state-of-the art statistical methods.
